# Bias Stability Investigation of a Triaxial Navigation-Compatible Accelerometer with an Electrostatic Spring

**DOI:** 10.3390/s22218102

**Published:** 2022-10-22

**Authors:** Decong Chen, Yanzheng Bai, Chengrui Wang, Shuchao Wu, Chunyu Xiao, Jianbo Yu, Zebing Zhou

**Affiliations:** MOE Key Laboratory of Fundamental Physical Quantities Measurement, Hubei Key Laboratory of Gravitation and Quantum Physics, PGMF, School of Physics, Huazhong University of Science and Technology, Wuhan 430074, China

**Keywords:** inertial navigation, bias stability, electrostatic accelerometer, electrostatic spring

## Abstract

The bias stability performance of accelerometers is essential for an inertial navigation system. The traditional pendulous accelerometer usually has a flexible connection structure, which could limit the long-term bias stability. Here, based on the main technologies employed in previous space missions of our group, we developed a terrestrial triaxial navigation-compatible accelerometer. Because there is no mechanical connection between the inertial test mass and the frame, the bias performance relies on the stability of the equivalent electrostatic spring, where further sources are analyzed to get the optimal electrostatic force scheme. To investigate the bias stability under different ranges, the vertical and horizontal measurement ranges are designed at 5 g and ±10 mg, respectively. A low-noise high-voltage levitation scheme is adopted to extend the vertical measurement range from sub-mg to more than earth’s 1-g gravity. Finally, the experimental validation results show that the 24-h bias stability of vertical and two horizontal directions come to 13.8 μg, 0.84 μg, and 0.77 μg, respectively.

## 1. Introduction

For long-term inertial navigation applications, gyroscopes and accelerometers with excellent bias stability are required. The accelerometer performance with scale factor stability of 1 ppm (parts per million) and bias stability of μg level are future trends [1,2]. The traditional accelerometers usually have a flexible connection structure between the test mass (TM) and the frame, influencing the bias stability. So different groups are attempting to improve bias stability by improving the mechanical stability [3] and the readout system [4]. Meanwhile, different kinds of accelerometers, such as micromachined accelerometers [5], optical accelerometers [6,7], quartz resonant accelerometers [8], vibrating beam accelerometers [9], hybrid quantum accelerometers [10], re-entrant cavity accelerometers [11], and so on, were developed to investigate the bias stability performance.

Electrostatic space accelerometers have been successfully employed in satellite earth’s gravity field recovery missions [12] and space gravitational experiments [13,14,15]. In the study of electrostatic accelerometers, more attention is paid to the noise characteristics of specific frequency bands [16]. Nevertheless, the electrostatic accelerometers potentially have better bias features for no mechanical connection between inertial TM and the frame. The bias stability depends on the equivalent electrostatic spring’s stability, where the temperature drift on the conventional physical linkage could be neglected. If the accelerometer’s performance, such as measurement range, bandwidth, and bias stability, is adjusted, it could be compatible with the requirements of inertial navigation.

In this paper, towards the bias stability with μg level, we developed a triaxial electrostatic accelerometer from space to terrestrial applications. A high-voltage levitation scheme is adopted to extend its vertical measurement range to overcome the earth’s gravity. The sources of bias stability from the electrostatic spring are analyzed. Moreover, an electrostatic scheme is designed and employed to optimize stability. Finally, the validation experiments show that the 24-h bias stability of vertical and two horizontal directions come to 13.8 μg, 0.84 μg, and 0.77 μg, respectively.

## 2. Principle and Design from Space to Ground

### 2.1. The Basic Approaches for Terrestrial Applications

**Figure 1:**
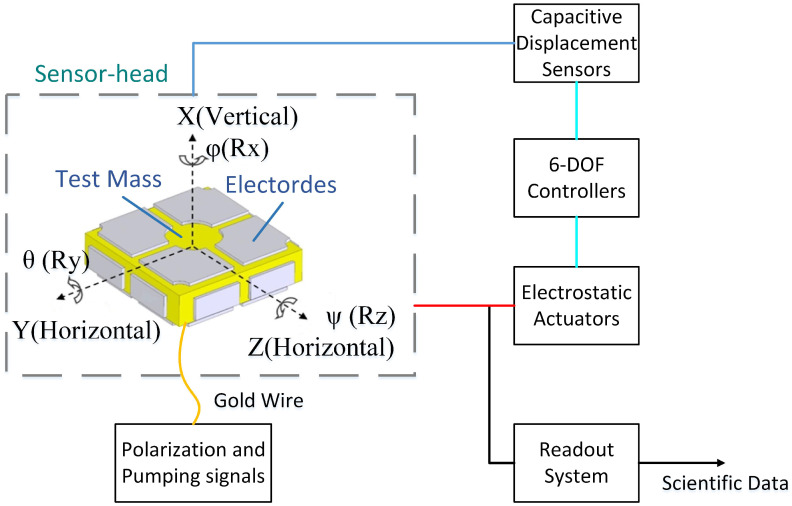
Systematic diagram of an electrostatic accelerometer, where the coordinate system, including three translational axes of vertical (X) and horizontal (Y and Z), and three rotational axes, are defined.

The basic schematic of a servo-loop electrostatic accelerometer, developed very early during space missions, is shown in Figure 1. It consists of a sensor head, a capacitive displacement sensor, a controller, a feedback actuator, and a readout system. The sensor head includes an electrode housing (EH) and a parallelepiped TM. The polarization voltage and the pumping signal are applied on the sensor head with a gold wire for the position sensors and electrostatic actuators. The sensor head is housed in a vacuum chamber with $10^{-5}$ Pa, so the impact of damping and dielectric inside the sensor head’s capacitor can be negligible. When the input acceleration changes, the position of the TM relative to the EH is changed, and this variation can be detected by the capacitive displacement sensors. Six servo-loops are used to maintain the TM motionless concerning the sensor cage. The measurements of the feedback voltages on the electrodes, which are proportional to the electrostatic forces or torques, provide the six outputs of the accelerometer.

The accelerometer translational feedback output $a_{f,i}$ can be expressed as

(1) $$a_{f,i}=H_{a,i}V_{f,i},$$

with

(2) $$H_{a,i}=\frac{2\epsilon S_{i}V_{b,i}}{md_{i}^{2}},$$

where $H_{a,i}$ is the actuator’s scale factor, $V_{f,i}$ is the feedback voltage applied on the electrodes for TM’s position control which is sampled as the scientific data, ϵ is the dielectric constant, $S_{i}$ is the effective electrode area, $V_{b,i}$ is the polarization voltage applied on the sensor head, *m* is the TM’s mass, and $d_{i}$ is the equilibrium gap between the electrode and the TM. Here, subscript i represents the X, Y, and Z axes.

According to (1) and (2), the main approaches to increasing the accelerometer’s measurement range are as follows:

- Increasing $V_{b,i}$ or $V_{f,i}$. This usually means that the high voltage amplifier circuit with several hundred volts output ability is necessary.
- Reducing $d_{i}$. Nevertheless, for present processing and installation technology, $d_{i}$ is more suitable in tens of microns to avoid high voltage breakdown caused by this small distance.
- Reducing *m* or increasing $S_{i}$. Lighter-density materials can be chosen, while hollow or sheet-shaped TM can also be considered to improve the area-to-mass ratio.

For terrestrial applications, the vertical measurement range must be over 1-g to balance the earth’s gravity. In this paper, the parameters of the sensor head remain nearly unchanged based on our previous manufacturing technologies [17]. So, a highly stable large $V_{b,i}$ and $V_{f,i}$ are mainly adopted to enlarge the range.

### 2.2. Bias Stability Analysis

Because of the accelerometer’s asymmetry, the TM’s actual control position is offset from its ideal position. This offset will couple with the equivalent electrostatic spring to produce the accelerometer’s internal bias [18]. The expression $a_{b,i}$ can be given by,

(3) $$a_{b,i}=\omega_{e,i}^{2}x_{b,i},$$

with

(4) $$\omega_{e,i}^{2}=\frac{\varepsilon S_{i}\left(V_{b,i}^{2}+V_{f,i}^{2}\right)}{md_{i}^{3}},$$

where $\omega_{e,i}$ is the equivalent angular frequency of the electrostatic spring, and $x_{b,i}$ is the TM’s actual position bias relative to its ideal equilibrium position.

The coupling between translational DOFs is mainly caused by the TM’s perpendicularity, which can be accurate to 10 arc-sec (about 50 μrad). Since the vertical range is much more extensive than the horizontal direction, its effect on the horizontal bias stability is much more significant than the coupling between horizontal DOFs. This coupling influence is not considered here but will be analyzed according to the prototype test results.

Then the absolute bias instability $\delta a_{b,i}$ can be given by,

(5) $${\left(\delta a_{b,i}\right)}^{2}={\left(\omega_{e,i}^{2}x_{b,i}\frac{\delta S_{i}}{S_{i}}\right)}^{2}+{\left(\omega_{e,i}^{2}x_{b,i}\frac{3\delta d_{i}}{d_{i}}\right)}^{2}+{\left(\frac{x_{b,i}a_{f,i}}{d_{i}V_{f,i}}\delta V_{b,i}\right)}^{2}+{\left(\frac{x_{b,i}a_{f,i}}{d_{i}V_{b,i}}\delta V_{f,i}\right)}^{2}+{\left(\omega_{e,i}^{2}\delta x_{b,i}\right)}^{2},$$

where $\delta S_{i}$, $\delta d_{i}$, $\delta V_{b,i}$, $\delta V_{f,i}$, and $\delta x_{b,i}$ indicate the absolute instability of the corresponding physical quantity. These bias stability sources can be classified into three parts: the sensor head’s mechanical performance (1st and 2nd items), the polarization and feedback voltages (3rd and 4th items), and the position offset (5th item).

The sensor head’s mechanical performance mainly depends on the thermal expansion coefficient of the sensor head and the ambient temperature stability. So ultra-low expansion glasses could be adopted as the material of the sensor head, whose thermal expansion coefficient is less than 1 ppm/K. If the temperature stability is controlled below 1K, the contribution of δdiδdididi and δSiδSiSiSi could be far less than 1 ppm, with $x_{b,i}\ll d{}_{i}$ which could be easily achieved by second-order nonlinearity effects compensation [19]. Moreover, the contribution of mechanical performance is less than that of the other items in (5).

To increase the measurement range for ground applications, the polarization voltage applied on the sensor head must reach about hundreds of volts. A commercial high precision reference voltage with a self-designed HV amplifying circuit, including an operational amplifier and an HV MOSFET, is used [20,21]. The stability of the HV amplifier circuit is further improved by selecting stable gain resistors with suitable values. Then the contribution of δVb,iδVb,iVb,iVb,i and δVf,iδVf,iVf,iVf,i can be theoretically controlled to less than 1 ppm.

The stability of the position offset relies on the capacitive position sensor. The output stability of the position sensor could achieve tens of pico-meters under the micro-meter level range [22,23,24]. So, the accelerometer bias contribution by $\delta x{}_{b,i}$ could be designed below 1 μg by coupling suitable equivalent electrostatic stiffness.

### 2.3. Optimal Scheme of Vertical Axis at Constant 1-g Input

**Figure 2:**
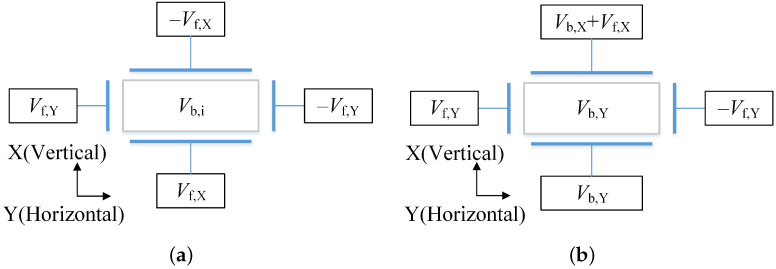
The electrostatic force scheme (**a**) on-orbit satisfies the relationship in (1), and the terrestrial scheme (**b**) to obtain optimal characteristics with 1-g input along the vertical direction.

The electrostatic force scheme of the space accelerometer is shown in Figure 2a, and the polarization voltage $V_{b,i}$ is applied on the TM shared by all 6 DOFs.

Due to the constant 1-g input, the polarization voltage in the vertical direction $V_{b,X}$ should be much higher than that in the horizontal directions $V_{b,Y}$ and $V_{b,Z}$. Meanwhile, to improve the accelerometer’s bias stability, a different electrostatic scheme is employed here.

According to (6), $\omega_{e,i}^{2}$ has a minimum value when $V_{b,i}$ is equal to $V_{f,i}$,

(6) $$\omega_{e,i}^{2}=\frac{\varepsilon S_{i}\left(V_{b,i}^{2}+V_{f,i}^{2}\right)}{md_{i}^{3}}\geq \frac{2\varepsilon S_{i}V_{b,i}V_{f,i}}{md_{i}^{3}}=\frac{H_{a,i}V_{f,i}}{d_{i}}=\frac{a_{f,i}}{d_{i}}.$$

where the accelerometer has minimal bias. Moreover, the position sensing stability ($\delta x{}_{b,i}$) has the most negligible effect on the bias stability of the accelerometer.

Due to generated by the similar HV amplifier, the noise of $V_{b,i}$ and $V_{f,i}$ has the same properties. $k_{\mathrm{HV}}$, the ratio of these voltages’ stability to output voltage is nearly the same, as shown as follows,

(7) $$\frac{\delta V_{b,i}}{V_{b,i}}=\frac{\delta V_{f,i}}{V_{f,i}}=k_{\mathrm{HV}}.$$

The item that is related to voltage stability also has a minimum value, as shown in (8)

(8) $${\left(\frac{\delta V_{b,i}}{V_{f,i}}\right)}^{2}+{\left(\frac{\delta V_{f,i}}{V_{b,i}}\right)}^{2}\geq 2\frac{\delta V_{b,i}}{V_{f,i}}\frac{\delta V_{f,i}}{V_{b,i}}=2k_{\mathrm{HV}}^{2}.$$

Regardless of the influence of mechanical structure and sensing performance, the electrostatic stiffness and its stiffness stability affected by voltages can achieve the minimum value synchronously when $V_{b,i}$ and $V_{f,i}$ are equal.

Therefore, the best $V_{b,i}$ for a vertical direction under constant 1-g input is given by,

(9) $$V_{b,X}=\sqrt{\frac{md_{X}^{2}a_{f,X}}{2\varepsilon S_{X}}}=\sqrt{\frac{md_{X}^{2}g}{2\varepsilon S_{X}}}\approx V_{f,X},$$

where the accelerometer has the slightest bias and the best bias stability. $V_{b,X}$ is obtained by amplifying a reference source by circuit, while $V_{f,X}$ is obtained by PID output. These two voltages are only numerically set relatively close but are independent signals.

According to Figure 2a and (9), the lower electrodes and the TM are equal potentials, and there is no electrostatic force between them. The upper electrodes generate all the electrostatic force to the TM to balance gravity. This electrostatic force condition is equivalent to the single-sided electrostatic force scheme, as shown in Figure 2b.

In this scheme, the horizontal Y and Z axis share the same polarization voltage applied to the TM for a similar measurement range. In the vertical direction X, since the measurement range is larger than the horizontal range, the vertical polarization voltage is significantly larger than that in horizontal directions. Therefore, the vertical polarization and feedback voltages are applied only to the upper electrodes. In contrast, the lower electrodes’ potential is equal to that of TM to achieve zero force between them. The vertical feedback output is shown in (10),

(10) $$a_{f,X}=\frac{\varepsilon S_{X}{\left(V_{b,X}+V_{f,X}\right)}^{2}}{2md_{X}^{2}}\approx g\;$$

in which the feedback voltage and acceleration are quadratic relationships. Although nonlinearity is introduced, the scheme could achieve optimal bias stability along the vertical direction.

### 2.4. Noise Model

**Figure 3:**
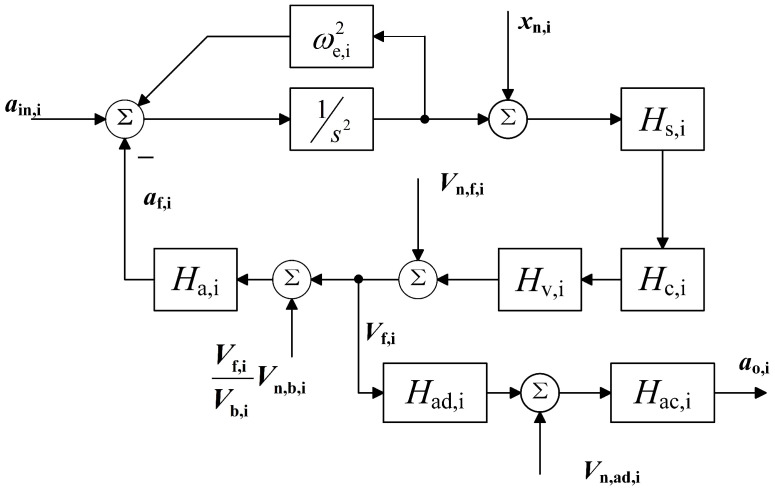
The noise model of the accelerometer.

The operational diagram and noise model of the accelerometer are shown in Figure 3 for ease of noise and stability assessment. In which *s* is the complex frequency. $H_{s,i}$, $H_{c,i}$, $H_{v,i}$, and $H_{a,i}$, respectively, represent the transfer function of the capacitive displacement sensing circuit, the control circuit, the HV amplifier circuit, and the actuator circuit. $a_{\mathrm{in},i}$ and $a_{f,i}$ are the input and feedback accelerations, respectively. The output acceleration is calculated with the output voltage $V_{o,i}$ and the actuator’s scale factor $H_{a,i}$, which is obtained by the prototype’s calibration. While $x_{n,i}$, $V_{n,f,i}$, $V_{n,b,i}$, and $V_{n,\mathrm{ad},i}$ are the noise introduced by the sensing, feedback, polarization, and readout circuits.

According to the noise model, the accelerometer’s output noise $a_{n,o,i}$ can be written as,

(11) $$a_{n,o,i}\approx \omega_{e,i}^{2}x_{n,i}+\frac{H_{a,i}}{1+H_{\mathrm{open},i}}V_{n,f,i}+\frac{H_{a,i}V_{f,i}}{V_{b,i}}V_{n,b,i}+H_{a,i}V_{n,\mathrm{ad},i}$$

in which $H_{\mathrm{open},i}$ is the open-loop gain.

The TM’s position readout circuit scheme, based on a capacitive-inductive resonant bridge, was settled very early during mission development. It comprises a preamplifier, a bandpass filter, and a demodulator. The sensing noise is mainly determined by the characteristics of the demodulator in the sensing circuit. The feedback voltage noise of the accelerometer is suppressed by $H_{\mathrm{open},i}$, while the reading circuit noise is affected by the ADC performance.

When there is an input acceleration, such as 1-g, $V_{f,i}$ will not be equal to 0. The polarization voltage noise is introduced into the accelerometer’s output as an out-of-loop signal. When there is no input acceleration outside, the influence of polarization voltage can be negligible ($V_{f,i}$ = 0), which is equivalent to the traditional accelerometer noise model.

According to Parseval’s theorem, the integration of power spectral density in the frequency domain and standard deviation in the time domain are equivalent. (5) and (11) have similar forms of expression. Therefore, the ultra-low frequency accelerometer noise model can estimate long-term stability.

### 2.5. Prototype Parameters Design and Performance Analysis

To verify the performance, especially the bias stability of this terrestrial triaxial accelerometer, an accelerometer prototype is design base on our previous space experiences. Here, an aluminum parallelepiped of 4 cm sides and 1 cm height is selected as the TM, whose mass is 44.64 g, which can be replaced with a gold-coated ceramic glass for similar density to improve the stability further. The polarization and maximum feedback voltages are over 300 V with the HV amplifier circuit along the vertical direction. Moreover, the single-sided electrostatic scheme mentioned in Section 2.3 was employed to achieve the best stability. The capacitance gap in the vertical direction is adjusted to 25 μm, so the range in this direction can be over 5 g.

For the horizontal direction, a different range is designed to compare the performance of the accelerometer relative to the vertical axis. The gap of the horizontal axis is designed at 50 μm, and the polarization and maximum feedback voltages are 60 V and 80 V, respectively. Therefore, the horizontal range can achieve about ±10 mg.

Due to the electrostatic stiffness, the accelerometer’s closed-loop bandwidth should be enough to achieve TM control. The bandwidth in the vertical direction is designed about 640 Hz, while in the horizontal direction is about 22 Hz. The accelerometer has good dynamic characteristics and can achieve motion measurements in these frequency bands. The control bandwidth can be adjusted accordingly if the range is further increased.

The main parameters of the accelerometer are listed in Table 1, and noise and stability are estimated based on the above analysis.

**Figure 4:**
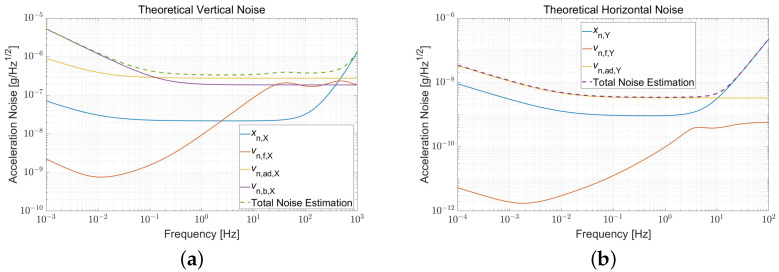
Theoretical noise estimation in (**a**) Vertical and (**b**) Horizontal directions, including the noise of the sensor circuit, HV amplifier circuit, and Readout system, which is the primary limitation of the total noise, are all converted to acceleration.

The theoretical noise floors of the accelerometer are about 0.35 $\mu g/\sqrt{\mathrm{Hz}}$ in the vertical direction and 3.3 n$g/\sqrt{\mathrm{Hz}}$ along the horizontal, respectively, as shown in Figure 4. According to Parseval’s theorem, bias stability can also be estimated by the intrinsic noise of the accelerometer. The estimated stabilities of the accelerometer are approximately 5 μg (Vertical) and 0.2 μg (Horizontal), respectively, in 24 h at a 10 Hz sample rate.

## 3. Experimental Investigation of the Prototype

### 3.1. The Prototype Instruments and Experimental Setup

The accelerometer prototype comprises a sandwich structure sensor head sealed in a vacuum chamber, three function electronic units (FEU), and one control electronic unit (CEU). The FEUs are used to realize the 6-DOF servo control, including a capacitive sensor, a controller, and a readout circuit. In contrast, CEU is used for pumping signal generation and data transfer between the prototype and a host computer.

**Figure 5:**
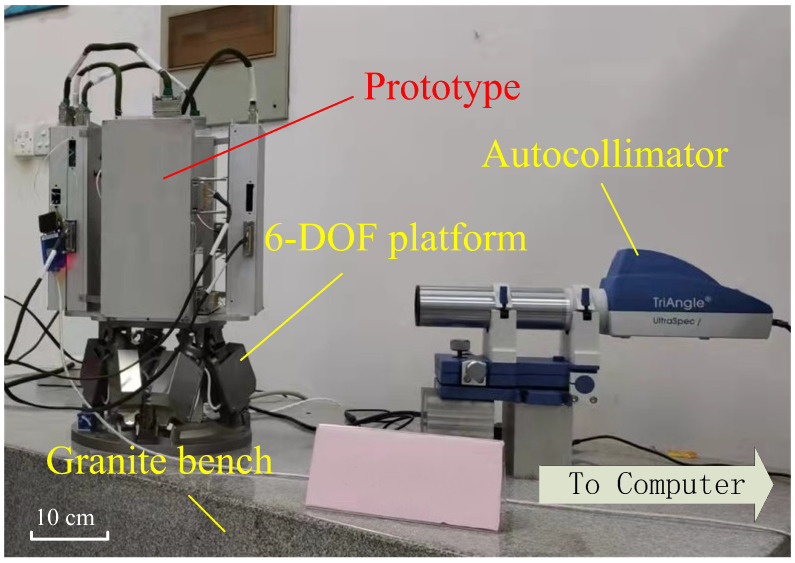
The instruments’ experimental setup includes the prototype, the positing platform, the autocollimator, and the granite bench.

The prototype is installed on a 6-DOF positing platform. Since the platform’s repeatability is only 2 μrad, an autocollimator with 0.25 μrad (0.05 arc-sec) accuracy is employed to monitor the attitude of the sensor head. All the instruments are placed on a granite bench to reduce the influences from seismic vibrations, as shown in Figure 5.

### 3.2. Hv Amplifier Test

According to the noise model, the non-zero feedback voltage will be coupled with the polarization voltage’s noise. This coupling will introduce an acceleration’s out-of-loop noise, which cannot be suppressed by $H_{\mathrm{open},i}$. Therefore, a stable polarization voltage is necessary to reduce this effect. The polarization voltage is generated by amplifying a reference voltage to tens or hundreds of volts.

The HV power supply is generated by a DCDC module, which can provide a maximum voltage of 1000 V to the circuit, and the power supply noise is about 4 $\mathrm{mV}/\sqrt{\mathrm{Hz}}$. Since the amplifier circuit’s power supply rejection ratio is over 120 dB at low frequency, the power supply’s influence is negligible. The HV output is tested and normalized to evaluate its characteristics, and the normalization result equals $k_{\mathrm{HV}}$ mentioned in Section 2.3.

**Figure 6:**
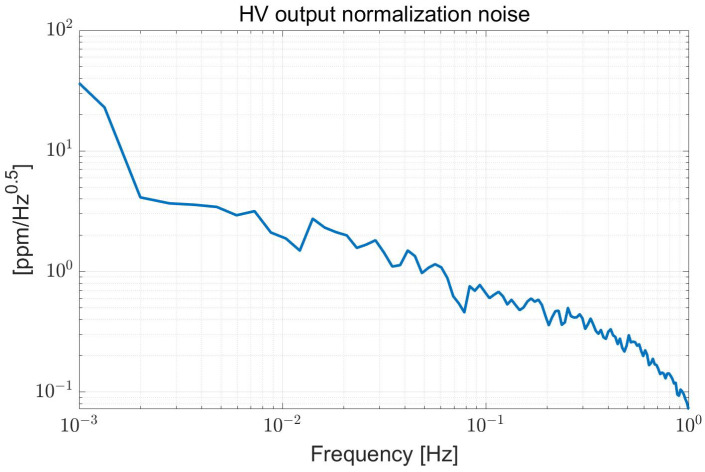
The normalization noise of HV output.

As shown in Figure 6, the normalization noise at 0.1 Hz is about 0.65 ppm$/\sqrt{\mathrm{Hz}}$, and the stability is about 0.8 ppm in the 30 min of data.

### 3.3. Servo-Control and Calibration of the Prototype

The prototype is initially adjusted with the platform. Both sensitive axes (Y and Z) are oriented with a specified tilt angle relative to the horizontal to reduce the gravity projection on the horizontal directions within the measurement range to achieve 6-DOF servo control.

**Figure 7:**
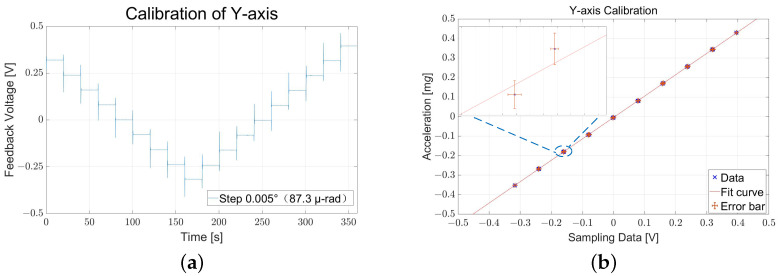
Scale factor and range calibration results of horizontal (Y). (**a**) The readout voltage variation with the adjustment of the platform. (**b**) Calibration result, in which “×” is the data used for the tilt test, while the zoomed-in picture shows error bars and test point differences introduced by the non-repeatability of the platform.

After the electrostatic compensation to suppress the influence of asymmetry, the scale factor calibration of the prototype is also carried out on the positing platform, with the sensitive axis adjusted between $-0.025^{\circ }$∼$0.025^{\circ }$ with respect to the horizontal (equivalent to applying a gravitational loading between −0.4∼0.44 mg) in 0.005${}^{\circ }$ steps monitored by autocollimator simultaneously, plotted in Figure 7a.

The tilt-induced gravitational loading to the Y-axis output is about 1.096 mg/V, while the Z-axis scale factor has a similar value of 1.043 mg/V. Since the operating range of the autocollimator is ±5 mrad, the platform data is utilized for the entire ±10 mg measurement range verification. And the test measurement ranges are about ±10.96 mg (Y) and ±10.43 mg (Z).

Since the maximum external input acceleration is only 1-g, the range along the vertical is estimated with the max upper electrodes’ voltage and the vertical equilibrium capacitance. The high voltage amplifier circuit can reach the maximum output of 690 V, so the max vertical electrostatic acceleration of the prototype is about 5.1 g, according to (10).

### 3.4. Noise and Bias Stability Test

**Figure 8:**
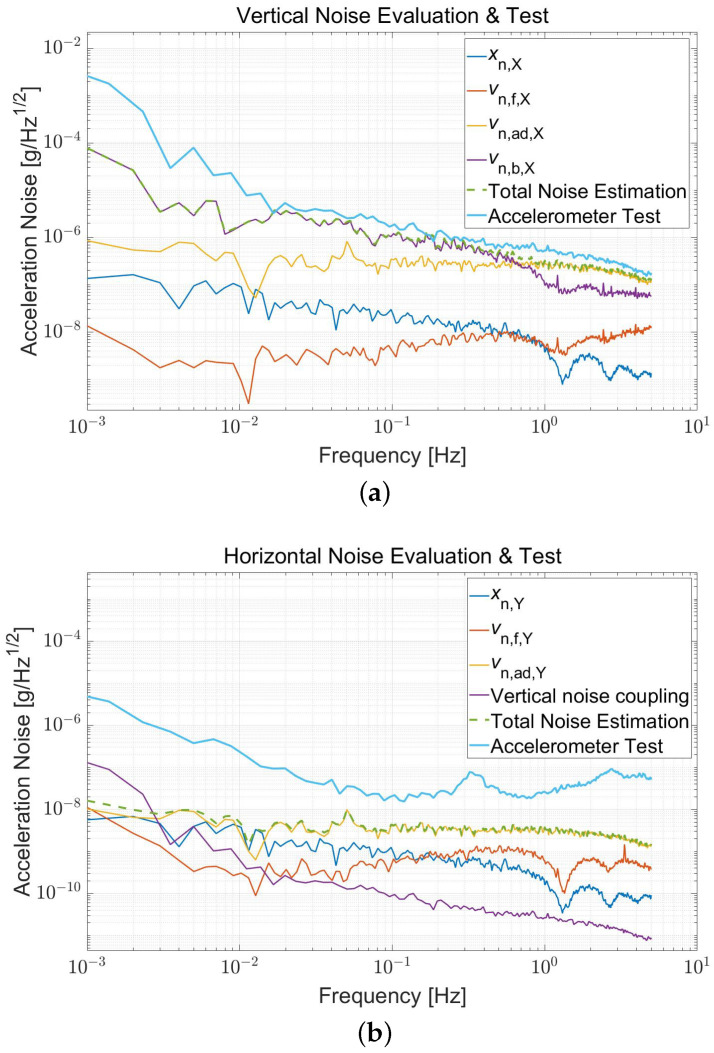
Total noise of the accelerometer estimation and test in the (**a**) Vertical and (**b**) Horizontal directions. By separately testing the noise of the sensing (Blue), high voltage feedback (Orange), ADC (Yellow), and polarization (**a**) or coupling noise (**b**) (Purple), the accelerometer noise floor (Green dashed line) is estimated according to (11). The prototype is tested to obtain its total noise (Light blue). The characteristic peaks of the seismic background are observed in the horizontal direction. Moreover, HV output along the vertical is introduced to the Horizontal direction with a coupling angle of 50 μrad. The high-precision multimeter’s lowpass filter causes the comb curve higher than 1 Hz.

The accelerometer sources have been independently tested before the prototype servo control and accounted for estimating the performance. The estimated noises, depicted as the sum of the capacitive sensor, HV Feedback, and readout system noise, are about 0.3 $\mu g/\sqrt{\mathrm{Hz}}$ and 4 n$g/\sqrt{\mathrm{Hz}}$, respectively, at 0.1 Hz along the vertical and horizontal, both primarily determined by the ADC characteristics. Figure 8 plots the total estimated (Green dashed line) and observed (Light blue) noise of the prototype.

The prototype is tested with a gravitational loading of 1-g (Vertical) and 0-g (Horizontal), and the digital output is utilized for noise measurement. The vertical noise is about 2 $\mu g/\sqrt{\mathrm{Hz}}$ at 0.1 Hz and 0.6 $\mu g/\sqrt{\mathrm{Hz}}$ at 1 Hz, respectively, nearly the same as the estimated noise. While the horizontal noise is about 20 n$g/\sqrt{\mathrm{Hz}}$ at 0.1 Hz, influenced by the ambient seismic background. In addition, cross-coupling of the vertical to the horizontal could come from the parallelism of the TM, which is less than 50 μrad, so even the worst crosstalk noise is only 10${}^{-10}$ g order of magnitude and could be negligible.

**Figure 9:**
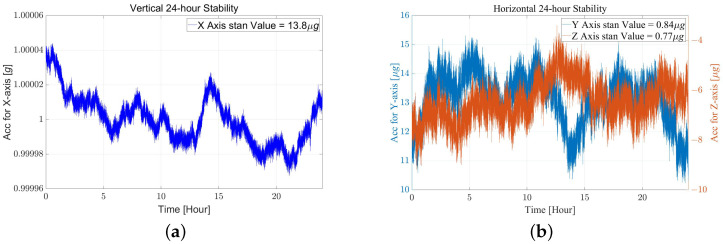
The 24-h bias stability test of the prototype with a gravitational loading of (**a**) vertical 1-g and (**b**) Horizontal 0-g, which is about 13.8 μg (X), 0.84 μg (Y), and 0.77 μg (Z), respectively.

Adjusting the platform until the horizontal output of the accelerometer is nearly 0, the output data’s standard deviation can be used to measure the bias stability. The prototype is tested for over 1 day at room temperature to assess the long-term bias stability, and the 24-h standard deviations, shown in Figure 9, are about 13.8 μg (Vertical X), 0.84 μg (Horizontal Y), and 0.77 μg (Horizontal Z), respectively.

**Figure 10:**
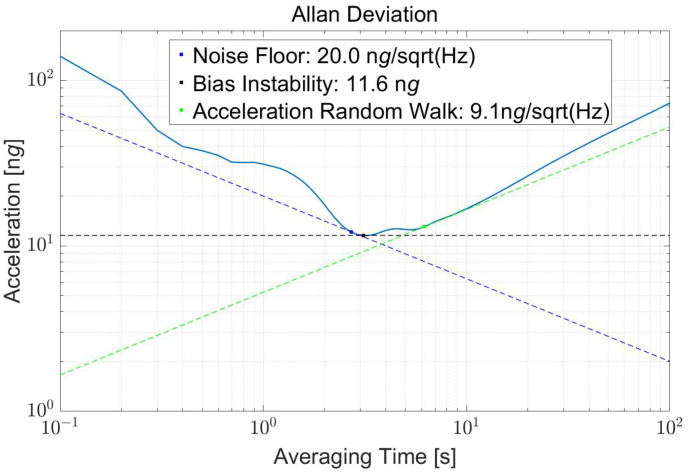
The vertical Allan deviation result, in which the noise floor is about 20 n$g/\sqrt{\mathrm{Hz}}$ and the bias instability is about 11.6 ng@3s.

Allan deviation is the most universally used figure of merit in sensor technology to quantify the instability of a device. However, limited by the ADC sampling rate in the vertical direction, only the horizontal result is shown in Figure 10. The bias instability is about 11.6 ng@3s, and the noise floor is the same as Figure 8.

### 3.5. Accelerometer Performance Summary

The summary of all the accelerometer’s performance test results is shown in Table 2.

The ratio of bias stability to the measurement range in the horizontal direction is about 80 ppm, and the value in the vertical direction is about 2.7 ppm. Meanwhile, the relative noise floors are about 2 ppm$/\sqrt{\mathrm{Hz}}$ (horizontal) and 0.4 ppm$/\sqrt{\mathrm{Hz}}$ (vertical).

The equilibrium gap *d* in the horizontal direction, twice as large as in the vertical direction, introduces more displacement instability with the same sensing circuit performance. The single-sided electrostatic scheme is employed in the vertical direction. This scheme could optimize stability while enabling a more extensive measurement range due to its quadratic relationships.

Although the vertical noise curves are relatively consistent, there are still differences in the horizontal direction. The horizontal result is affected by ground vibrations, and seismic peaks can be observed obviously in Figure 8. Meanwhile, the effects of the demodulator (such as phase noise) cannot be estimated and coupled with electrostatic stiffness. Therefore, the sensing noise will be greater than the estimated result and cannot be suppressed by open-loop gain. Temperature control is not employed, and the positing platform is more affected by temperature. Hence, a long-period drift occurs in the horizontal directions and will influence long-term stability.

## 4. Conclusions and Discussion

In this paper, based on the advantages of no mechanical connection between the TM and EH in space electrostatic accelerometer, a triaxial accelerometer for terrestrial applications is developed by increasing the vertical measurement range. An optimal electrostatic force scheme is analyzed and designed to estimate the best result of this type of accelerometer. The bias stability of the accelerometer has been analyzed and tested. The main results show that the measurement ranges are about 5.1 g (Vertical) and ±10 mg (Horizontal). Meanwhile, the 24-h bias stabilities are about 13.8 μg (Vertical X), 0.84 μg (Horizontal Y), and 0.77 μg (Horizontal Z), respectively. At 0.1 Hz, the vertical and horizontal noise floors are about 2 $\mu g/\sqrt{\mathrm{Hz}}$ and 20 n$g/\sqrt{\mathrm{Hz}}$, respectively. The relative noise floor and bias stability in the vertical direction achieved 0.4 ppm$/\sqrt{\mathrm{Hz}}$ and 2.7 ppm. So the current prototype could be compatible with a gimbaled inertial navigation system, but it needs the inertial platform within the accelerometer’s measurement range.

Meanwhile, there is still a particular gap between the prototype and the QA 3000 [25], a wide-range inertial navigation grade accelerometer whose relative noise floor and bias stability are about 0.04 ppm$/\sqrt{\mathrm{Hz}}$ and 0.67 ppm. The prototype’s performance can be improved as follows.

Firstly, the measurement range of the horizontal axis could be further increased to 1 g by optimizing the sensor head’s parameters, such as reducing the TM’s mass and the gap, and so on. However, a high surface-to-mass ratio reduces the TM’s specific stiffness and fundamental frequency mode, making it prone to deformation during processing and transportation. While significant potential differences at a small gap can cause the electrodes to break down. Therefore, the acceleration measurement range’s limitations should be analyzed in the future. A measurement range that can be achieved is estimated here. For example, changing the mass block to a hollow cube with a side length of 4 cm will only slightly increase its mass. With the same current circuit and sensor head’s geometry in the vertical direction in Table 1, the triaxial range of the accelerometer can be greater than 4 g.

Secondly, according to the analysis in Section 2.2, the accelerometer’s bias mainly comes from electrostatic stiffness coupling with the sensing position’s accuracy. This article presents a scheme for the minimum electrostatic stiffness in Section 2.3. However, due to the negative electrostatic stiffness, the accelerometer’s system is not stable. The sensing factor is designed for a full-range measurement of TM’s position. The position accuracy, affecting the accelerometer’s noise and bias stability, is limited by this small sensing gain, which should be increased. In addition, according to the accelerometer’s modified noise model in Section 2.4, the polarization voltage’s stability also affects the accelerometer’s performance when the input acceleration exists. Furthermore, if better stability wants to be achieved, the position accuracy of the sensing and the stability of polarization voltage should be further improved.

Finally, the temperature coefficient of the 6-DOF platform and the ground vibration affect the feature test, so temperature control and vibration isolation systems should be employed to evaluate the accelerometer’s performance accurately.

This work is a preliminary attempt to investigate the terrestrial application of space accelerometers. Therefore, this accelerometer may be potentially applied for inertial navigation, gravity, tilt, and seismic measurement in the future.

## Figures and Tables

**Table 1:** The general parameters of the accelerometer.

Parameter	Vertical	Horizontal
$d_{i}$	25 μm	50 μm
$S_{i}$	930 mm${}^{2}$	130 mm${}^{2}$
$V_{b,i}$	300 V	60 V
Max $V_{f,i}$	300 V	80 V
Max $a_{f,i}$	5 g	±10 mg
$\omega_{e,i}^{2}$	$8\times 10^{5}/s^{2}$	$1583/s^{2}$
Bandwidth	640 Hz	22 Hz
$\delta x_{b,i}$ *	<60 pm	<1 nm
$\delta a_{b,i}$ *	<5 μg	<0.2 μg
$a_{\mathrm{noise},i}\left(f\right)$	0.4 $\mu g/\sqrt{\mathrm{Hz}}$	3 n$g/\sqrt{\mathrm{Hz}}$

* This stability is estimated with a case of 24-h and 10 Hz sample rate.

**Table 2:** Prototype’s performance summary.

Experimental Performance	X (Vertical)	Y (Horizontal)	Z (Horizontal)
Range	5.1 g	±10.96 mg	±10.43 mg
Scale factor	0.323 g/V	1.096 mg/V	1.043 mg/V
Tested noise PSD	2 $\mu g/\sqrt{\mathrm{Hz}}$	20 n$g/\sqrt{\mathrm{Hz}}$	20 n$g/\sqrt{\mathrm{Hz}}$
24-h Bias stability	13.8 μg	0.84 μg	0.77 μg
Bias instability	/	11.6 ng	12.0 ng

## Data Availability

Not applicable.
